# The impacts of global economic policy uncertainty on green bond returns: A systematic literature review

**DOI:** 10.1016/j.heliyon.2024.e25076

**Published:** 2024-01-24

**Authors:** Samuel Asante Gyamerah, Clement Asare

**Affiliations:** Department of Statistics and Actuarial Science, Kwame Nkrumah University of Science and Technology, Ghana

**Keywords:** Systematic literature review, Green bond, Sustainable finance, Economic policy uncertainty, Financial markets, Spillover index, Risk management, Climate change, Preferred Reporting Items for Systematic Reviews and Meta-Analyses (PRISMA)

## Abstract

This study utilizes the Preferred Reporting Items for Systematic Reviews and Meta-Analyses (PRISMA) framework to investigate the interconnectedness of green bond with various financial markets, aiming to clarify their relationship with global economic uncertainty and their impact on returns. After a comprehensive search of pertinent research papers from January 2016 to September 2023, 79 relevant articles were identified. The analysis delves into the evolution of research on green bonds' interactions with economic policy uncertainty considering the financial markets, analytical methodologies, contributions to the field, and the role of green bonds under both normal and extreme market conditions. The study reveals noteworthy findings: firstly, the interplay between green bonds and financial markets is influenced by macroeconomic factors, such as the COVID-19 pandemic and the Russia-Ukraine conflict in 2022, which were significant sources of economic policy uncertainty during the study period. Secondly, during times of global economic uncertainties, green Bonds act as net transmitters of spillovers in the short term but shifts to net receivers in the long term, positioning them as strategic hedging assets rather than safe-havens, particularly against spillovers from crude oil and CO2 emission in times of economic uncertainties. Additionally, the review highlights prevalent methodologies employed to assess the relationship between global economic policy uncertainty and green bonds. Some of which include quantile approaches, the Diebold & Yilmaz 2012 spillover index, as well as various models like VAR models, GARCH models, ARDL models. Notably, certain countries like China, the United Kingdom, and Vietnam emerge as key contributors to this research domain. The review not only consolidates existing knowledge but also provides valuable insights for investors and policymakers regarding green bonds in terms of risk management and asset allocation, while also pointing towards potential avenues for future research in this field.

## 1 Introduction

Throughout the past century, the global economy's overall stability has been tried and tested innumerable times. There has never been peace in the global economy, which stretches back to occurrences like the Great Depression of 1929-1939, the OPEC Oil Price Shock of 1973, the Asian Crisis of 1997, and without going too far, the Russian invasion of Ukraine in 2022. These persistent turbulence has given rise to a concept known as economic policy uncertainty (EPU), which arises when governmental policies and regulatory frameworks lack clarity in the market [4], [22]. The presence of economic policy uncertainty (EPU) casts a long shadow over the global financial markets [17], [59], [15], influencing them in various ways. This uncertainty can lead to heightened market volatility [50] and risk aversion among investors [58]. When governments introduce unclear or inconsistent policies, investors become uncertain about the future economic landscape, leading to a reluctance to commit capital to long-term investments. As a result, stock markets may experience increased fluctuations, and investment decisions can become more erratic. The role of green bonds emerges as a promising avenue for stability and sustainability [5], [26]. With the introduction of green bonds during a period of global policy uncertainty, an extensive review is required to identify the contributions and research focus in order to direct future research in times of need. First and foremost, what exactly is a green bond? Green bonds can be used with any bond format, including use-of-proceeds bonds (also known as plain vanilla bonds), project bonds, securitized bonds (ABS), and so on [12]. Unlike conventional bonds, green bonds are a highly encouraging market-driven approach for directing finances toward environmentally beneficial initiatives and for increasing awareness of environmental risks, offering a degree of predictability and purpose in an otherwise uncertain economic environment [18].

Regarding how investment decisions can be linked to green bonds, in the timeframe spanning from 2014 to 2019, Pham and Huynh [38] identified a connection between investor focus, as quantified by the Google Search Volume Index, and the green bond market performance, gauged through five green bond market indices. Their findings indicated a significant correlation between investor attention and both the returns and volatility of the green bond market. Specifically, they noted that this relationship fluctuates over time, displaying greater strength in the short term compared to the long term. This findings was also confirmed in a study by [16]. As a result, many studies have been carried out to investigate whether green bonds serve as a financial hedge or a safe asset for investors [20], [35]. During times of economic policy uncertainty (EPU), investors often seek refuge in assets that are perceived as more resilient and aligns with long-term sustainability goals. Due to green bond's ability to satisfy both the financial and environmental needs of investors, it presents possible avenues for risk management and diversification [24]. However, the growing unpredictability of economic policies is certain to have repercussions on financial markets, and green bonds are not exempt from this influence [51], [17]. This, in turn, has stimulated research examining the relationship between green bonds and different financial assets [9], [40], [16], especially in periods of economic policy uncertainties. Therefore, as concerns about uncertainty in economic policy persist, it has become increasingly important to understand how this uncertainty relates to the performance of green bonds.

### 1.1 Relationship between economic policy uncertainty and global financial markets

A study on how economic policy uncertainty affects stock markets globally was carried out by Al-Thaqeb and Algharabali [3]. The findings of their research demonstrated that policy uncertainty exerts a notable influence on both corporate financial strategies and consumer expenditure. More precisely, businesses tend to adopt a more cautious approach when confronted with elevated levels of uncertainty. Economic policy uncertainty (EPU) has been studied in relation to market-driven common stock returns as well as individual-driven idiosyncratic stock returns by Liao et al. [29]. They also looked into how this relationship was affected by corporate environmental responsibility (CER) activities. Based on a sample of 175 companies listed between 2008 and 2016 that were included in the Shanghai and Shenzhen 300 index, their study was conducted. According to the study's conclusions, an increase in EPU causes market-driven common stock returns to significantly decline, but it has the reverse impact, raising returns on individual-driven idiosyncratic stocks. Additionally, Fang et al. [17] investigated the long-term correlation between U.S. stock markets and crude oil prices. They specifically looked at the relationship between this dynamic correlation and the Economic Policy Uncertainty (EPU) score. They used the DCC-MIDAS model to perform their analysis, and the results showed that the EPU index had a considerable beneficial impact on the long-term correlation between the stock and oil markets. Wu et al. [53] looked at the time-frequency connection between the financial and commodity markets while taking economic policy uncertainty (EPU) into consideration. They did this by combining a rolling window technique with the DY and BK approaches to investigate how this relationship changes at different frequencies and across time. Their empirical results demonstrate that the financial and commodity markets exhibit powerful short-term information transmission, heightened volatility, and risk transmission, particularly during times of crisis. Chai et al. [10] investigated the dynamic nonlinear relationships between green bonds, stock prices, and clean energy during the global COVID-19 pandemic using weekly data from July 1, 2011, to July 9, 2021. For their analysis, they used a time-varying parameter vector autoregression model (TVP-VAR). According to their findings, the medium-term detrimental impact of green bonds on stock prices was mitigated by the COVID-19 epidemic. Based on their impulse response analysis, they also found that stock prices typically increased after a positive shock to renewable energy. Their analysis indicated that this beneficial effect was most noticeable during the period of economic recovery. Similar research was done on the impact of oil prices on the performance of green bonds in a recent work by Su et al. [46]. They looked at the relationships between oil prices and the green bond index from January 2011 to November 2021 using the quantile-on-quantile (QQ) approach. The study's conclusions show that the green bond index is positively impacted by oil prices in the near run. This implies that the market for green bonds may grow in response to higher oil prices, suggesting that green bonds could be a useful tool for reducing the impact of oil price shocks. Lin and Su [30] examined the relationship between three uncertainty indicators—financial, oil market, and economic policy—and the tail dependency of the green bond markets in the United States and China. In order to capture predictive causal links, they performed the quantile causality test for robustness and utilized a novel cross-quantilogram method to address this problem. The results of the study showed that the three chosen uncertainty indicators are important predictors of volatility and returns in green bond markets. These metrics, meanwhile, have different functions in the two countries. In particular, financial uncertainty is the most significant factor influencing US green bonds, whereas economic policy uncertainty is more significant in the case of China. Using a wavelet-based quantile dependence methodology, Wei et al. [51] investigated the association between Economic Policy Uncertainty (EPU) and green bond markets from 2014 to 2021. Their findings indicate that the relationship between EPU and the green bond market is a non-linear, time-varying Granger causality. A recent study by Peng et al. [37] investigated how economic policy uncertainty (EPU) affects green innovation. They employed the panel fixed effects model, analyzing data from 2000 to 2017, encompassing 31 Chinese provinces. Their main finding suggests a positive connection between EPU and green innovation, with noteworthy variations in the influence of EPU across provinces, depending on their levels of marketization and trade openness.

### 1.2 Analyzing the role of green bonds in times of economic policy uncertainty

The dynamic interrelationships between global rare earth elements, green bonds, clean energy stocks, and economic policy uncertainty were examined by Haq et al. [20]. To examine the co-movement of these indices over time, they utilized a dynamic conditional correlation-multivariate generalized autoregressive conditional heteroscedasticity (DCC-MGARCH) model. According to their research, when economic policy is uncertain, green bonds typically act more as a hedge than as a haven. Saeed et al. [42] conducted a thorough analysis on how well clean and green assets can be used to hedge against two forms of dirty energy assets, namely crude oil prices and an energy exchange-traded fund (ETF). They collected daily data spanning from January 3, 2012, to November 29, 2019, and employed corrected dynamic conditional correlation models for their analysis. The findings of their research indicated that it is advisable for investors to employ a dynamic hedging strategy. Examining how the COVID-19 affected the connection between Green Bonds and various financial assets, Naeem et al. [35] conducted a study that covered the global stock market, bond market, oil, USD index, and two common hedging options (Gold and Bitcoin) from May 2013 to August 2020. They followed the methodologies of Diebold and Yilmaz [13] and Baruník and Křehlík [6] and then calculated the hedge ratios and hedge effectiveness of green bonds for the financial assets considered for their research. Their results suggested that financial assets may have varying relationships with green bonds. Despite being in its early stages, the study implied that green bonds should not be overlooked during crises, as they can act as hedges for some assets but could possibly worsen the spread of negative impacts during those periods. To further evaluate the timing when the hedging ability becomes evident, Elsayed et al. [16] conducted a study to explore the relationship between green bonds and financial markets. They used a multivariate wavelet approach and combined Ensemble Empirical Mode Decomposition (EEMD) with the Diebold and Yilmaz [13] spillover framework to analyze dynamic connectedness. The results showed that diversification benefits were more pronounced in the short term, and in the long term, green bonds and financial markets exhibited strong integration. However, Saeed et al. [42] concluded in their study that, when it comes to hedging, clean energy stocks outperform green bonds, particularly in relation to crude oil. Nevertheless, Wang et al. [50] study's findings revealed both positive and negative effects of crude oil prices on the green bond index within the same sample when they conducted a study that explored the relationship between crude oil prices and green bonds. Specifically, high crude oil prices were associated with positive impacts, suggesting that an increase in crude oil prices could positively influence the green bond market. This indicates that green bonds can remain resilient even when faced with uncertainties that relate to crude oil prices. A recent investigation by Adekoya et al. [1] aimed to uncover the underlying reasons for the performance of green bond markets. They analyzed data encompassing commodity and financial asset prices, alongside speculative factors, to make projections regarding green bond returns. The Feasible Quasi-Generalized Least Squares (FQGLS) and causality-in-quantiles estimators were employed for this purpose. The outcomes indicated that most factors significantly predicted green bond returns. Speculative factors were found to have an adverse predictive impact, while commodity and financial asset prices exhibited a varied predictive influence. Furthermore, all factors, except for investor sentiment, were observed to influence green bond returns across different market scenarios.

Although there is a growing body of research on green bonds and economic policy uncertainty, a thorough evaluation that identifies potential research gaps and directions has not yet been conducted. Our goal in this research is to conduct a systematic literature review on the relationship between green bonds and the unpredictability of global economic policy. In order to review the relationship between global economic policy uncertainty and green bond returns, this study will examine the following topics: the areas of greatest interest, the countries making significant contributions, the key authors and their contributions, trends in publications on this topic, and the methodologies used in prior studies. By shedding light on the current state of knowledge, the study will help investors and policymakers make educated decisions as they navigate the complex relationship between uncertain economic policies and sustainable financing. It will also serve as a potential research target for scholars. Our review specifically aims to address a number of important topics. 1) we want to comprehend the nature of the connection between returns on green bonds and the unpredictability of economic policy. 2) we want to pinpoint the main lines of inquiry and possible gaps in our knowledge regarding this relationship. 3) we look at the approaches that have been used in previous research and assess their strengths and weaknesses. 4) We also explore a few prominent authors and their contributions to this area. 5) Finally, we investigate the most interesting sub-fields of this field of study.

Our research is significant because it fills an essential void in the present body of knowledge regarding the relationship between green bonds and the uncertainty surrounding global economic policies. Our study intends to explain the complexities of this relationship, suggest areas where additional research is needed, critically examine previous techniques, and highlight the significant contributions of prominent researchers in this subject through the thorough implementation of a systematic literature review. Our study's findings will not only be useful to investors and legislators in their decision-making processes, but will also present researchers with a viable route for future research. Furthermore, our research contributes considerably to our understanding of sustainable financing in the context of volatile economic policies, laying the groundwork for more successful and long-lasting financial solutions. The other parts of the document are organized as follows: Section 2 outlines the methodology used for this review, encompassing details on the search database and selection criteria. In Section 3, a comprehensive review and extensive discussion are provided. Section 4 presents a discussion of the review. Lastly, Section 5 concludes the systematic literature review and presents some recommendations.

## 2 Methodology

### 2.1 Methodological approach, search strategy and selection process

**Figure 1:**
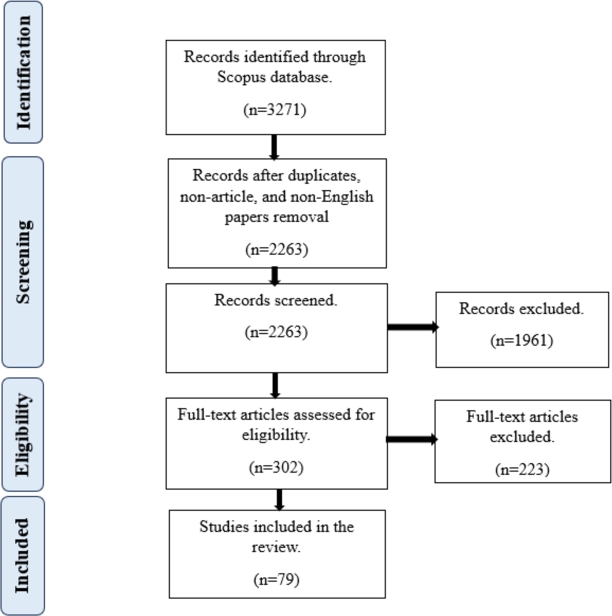
The systematic review methodology.

In accordance with the PRISMA (Preferred Reporting Items for Systematic Reviews and Meta-Analyses) framework, our systematic literature review applied a robust set of inclusion and exclusion criteria. We conducted a comprehensive search in Scopus, covering the period from January 1, 2016, to September 10, 2023, resulting in a total of 3,271 search results. We began our inquiry on January 1, 2016, despite the relationship between economic policy uncertainty and green bonds becoming apparent in 2019 (see Table 1). The inception of green bonds dates back to 2008 [28], encouraging us to examine the few years that came before the intersection with economic policy uncertainty in order to gain a comprehensive understanding of this upswing. Also, the time period concluded on September 10, 2023, as the search was performed on the same date. We selected Scopus as our database of choice because of its broad coverage, making it ideal for in-depth exploration of specialized research areas [36]. Scopus contains 34.02% more indexed articles than Web of Science [43], further enhancing its suitability for our study. Duplicate records and non-article documents were subsequently removed, leaving us with a refined set of 2,275 articles. We further filtered the dataset by excluding articles not published in English, resulting in 2,263 articles. To maintain a tight focus on the central theme of our study, we systematically excluded papers that did not pertain to the intersection of “Policy Uncertainty” and “Green Bonds,” culminating in a set of 302 articles. In our commitment to open access accessibility, we retained only those articles designated as “open access,” ultimately yielding a final selection of 109 papers. After reading the abstract of the 109 papers, we excluded 30 papers which were not relevant for the studies, leaving us with 79 papers for our systematic review. This rigorous selection process ensured the incorporation of high-quality, pertinent, and readily accessible studies for our review. For reproducible results, this was the search used in Scopus “policy AND uncertainty OR global AND policy AND uncertainty AND green AND bond OR green AND bond AND returns AND PUBYEAR > 2015 AND PUBYEAR < 2024 AND (LIMIT-TO (OA, “all”)) AND (LIMIT-TO (DOCTYPE, “ar”)) AND (LIMIT-TO (LANGUAGE, “English”)) AND (LIMIT-TO (EXACTKEYWORD, “Green Bonds”) OR LIMIT-TO (EXACTKEYWORD, “Economic Policy Uncertainty”) OR LIMIT-TO (EXACTKEYWORD, “Green Bond”) OR LIMIT-TO (EXACTKEYWORD, “Green Finance”))”. Fig. 1 illustrates the PRISMA framework, detailing the inclusion and exclusion criteria leading to the final number of papers in our systematic review. It is important to note that our findings are subject to significant methodological constraints. Specifically, the PRISMA framework, while valuable in its structured approach, has limitations that impact the broader applicability of our results. Notably, the exclusion of non-published articles by the PRISMA framework is a noteworthy limitation. This constraint could potentially influence the comprehensiveness of our findings by not considering relevant information available outside the published literature.

**Table 1:** Publications by year from 2016 to 2023.

Year	Publications
2016	0
2017	0
2018	0
2019	2
2020	3
2021	11
2022	26
2023	37

## 3 Results, analysis and discussions

### 3.1 Results and analysis

In this section, several variables are analyzed to provide a clear picture of the topic. Keyword co-occurrence is employed to identify prevalent themes and trends in the literature, offering insights into key concepts related to the impacts of global economic policy uncertainty on green bond returns. We also analyze contributions by author names to gauge scholarly influence within this context. The examination of top journals is also assessed to provide insights into the academic platforms shaping the discourse and assess paper quality on the topic. Additionally, the exploration of publications by country and subject area helps uncover geographical and thematic variations in research focus. Furthermore, the analysis of publications by year is also considered to allow us to trace the evolution of the literature over time, bringing attention to emerging trends and shifts in research emphasis. Finally, we conduct a comprehensive review of various methodologies used to assess the impact of economic policy uncertainty on green bond returns.

#### 3.1.1 Keyword co-occurrence network analysis of the selected papers for the systematic review

**Figure 2:**
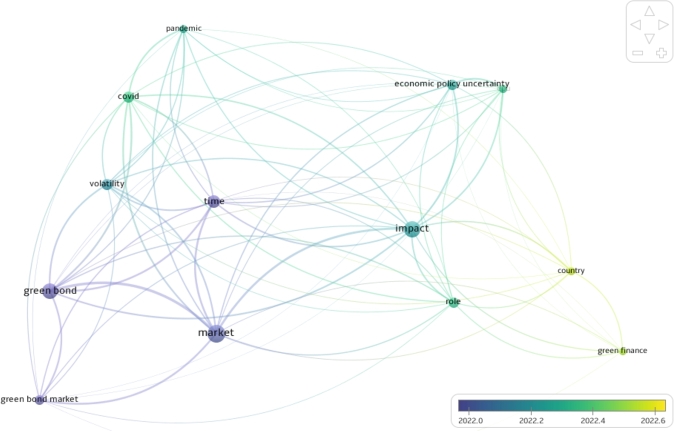
Keyword co-occurrence network analysis.

Fig. 2 presents the Keyword Co-occurrence Network Analysis of the Selected Papers for the Systematic Review analyzed with the VOSviewer software. From Fig. 2, it is evident that the color bar in VOSviewer, which spans from early 2022 (2022.0) to mid-2022 (2022.6), plays a crucial role in depicting a significant shift in research focus. The transition from blue to yellow along this color gradient is particularly noteworthy, as it reflects a temporal shift in research emphasis. Notably, this shift can be linked to the Russia-Ukraine war, which commenced in February 2022. The changing color along the color bar suggests that, as the year progressed, researchers began to pay increasing attention to the cross-country impact of economic policy uncertainty and green bonds, especially in relation to the role of green finance. The yellow connection lines between “country” and “green finance” indicate that these concepts gained prominence during the specified period.

#### 3.1.2 Publication by year

The total number of papers published on the topic within the study period is presented in Table 1, revealing a notable shift in research interest and publication activity in recent years, particularly with regard to the intersection of global economic policy uncertainty and green bond returns. It is striking to observe that there were no publications on this topic from 2016 to 2018, indicating a limited scholarly focus during that period. However, a significant uptick in research emerged from 2019 onward, aligning with global policy events, most notably the outbreak of the COVID-19 pandemic in 2020. This surge in publications suggests that the unprecedented economic challenges posed by the pandemic prompted scholars to explore the relationship between economic policy uncertainty and green bond performance. With 11 publications in 2021, and a remarkable 26 and 37 publications in 2022 and 2023 respectively, it is evident that the research community is increasingly recognizing the importance of understanding how global economic policy uncertainty influences the dynamics of green bond markets. This growing body of literature underscores the urgency of comprehending the roles green bonds can play in a world characterized by heightened policy uncertainties, potentially serving as a means to channel investments towards sustainable and climate-friendly projects even during turbulent economic times.

#### 3.1.3 Contributions by subject area

**Figure 3:**
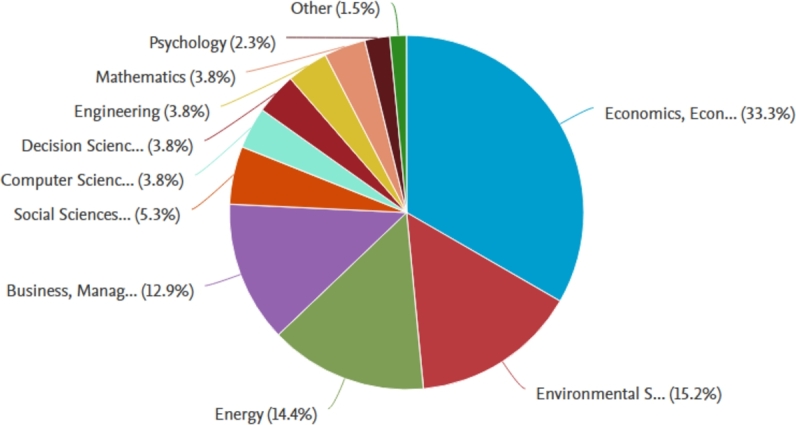
Papers published by subject distribution.

The breakdown of papers by subject area (see Fig. 3) in this systematic literature review highlights the predominant focus on Economics, Econometrics, and Finance, with a substantial 33.3% of the total papers falling within this category. This emphasis underscores the paramount importance of understanding the economic dimensions and financial implications of global economic policy uncertainty on green bond returns. Following closely, the Environmental Science area comprises 15.2% of the papers, indicative of the growing recognition of the environmental aspects associated with green bonds and their intersection with broader global policy uncertainties. The distribution of the remaining papers, as shown in Fig. 3, signifies a diverse range of subject areas being explored in the context of this research topic, suggesting a multidisciplinary approach to comprehending the complex dynamics between global economic policy uncertainty and green bond performance.

#### 3.1.4 Publications by country

**Figure 4:**
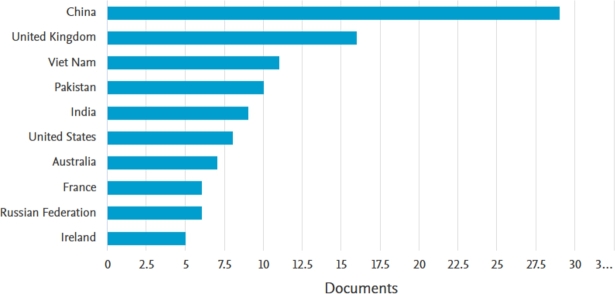
Countries with highest contribution.

The distribution of papers by countries in this systematic literature review as shown in Fig. 4 reveals that China takes the lead with 29 papers, signifying its growing prominence in both green finance and global economic policy discussions. The United Kingdom follows with 16 papers, reflecting their active contributions in this area. Additionally, Vietnam has made a significant contribution to this body of knowledge with 11 papers. Fig. 4 further illustrates the participation of other countries in this research endeavor. These results highlight the global nature of the subject matter and the recognition of its relevance by scholars from various regions.

#### 3.1.5 Top authors by citations and contributions

**Figure 5:**
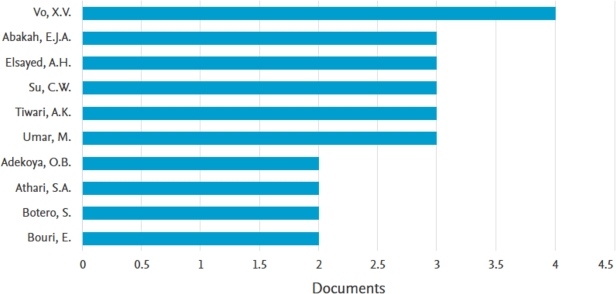
Contributions by authors.

The analysis of the leading authors in the domains of global economic policy uncertainty and green bond research is presented in Fig. 5 and Table 2. According to the data presented in Fig. 5, it is evident that Vo, X.V is a significant contributor in the field, having authored four publications. Following closely behind are Abakah E.J.A, Elsayed A.H, Su C.W, Tiwari A.K, Umar, M, each of who has three papers attributed to their names. Fig. 5 illustrates the significant contributions made by other prominent authors, highlighting their considerable engagement in this particular topic. Nevertheless, it is important to acknowledge that the publication by Le et al. [28] in the journal Technological Forecasting and Social Change has garnered the highest number of citations, amounting to 147. Similarly, the research conducted by Yu et al. [57] in the field of Energy Economics has received significant attention, with an impressive total of 140 citations, as depicted in Table 2. Abakah Emmanuel Joel Aikins and Tiwari Aviral Kumar, two highly prolific authors in terms of scholarly contributions, were among the co-authors of the study in [28]. This collaboration highlights the potential collaboration between prolific researchers and the production of influential publications. Moreover, the inclusion of Elsayed A.H and Tiwari A.K as co-authors in numerous prominent scholarly articles signifies their combined efforts in generating exceptionally impactful research. The close collaboration between highly productive authors and their participation in highly cited publications underscores the crucial significance of collaboration and specialized knowledge in furthering our comprehension of the complex interplay between global economic policy uncertainties and green bonds.

**Table 2:** Top authors, sources, and citations.

Author	Source	Citations
Le et al. [28]	Technological Forecasting and Social Change	147
Yu et al. [57]	Energy Economics	140
Sinha et al. [44]	Journal of Environmental Management	120
Tiwari et al. [47]	Global Finance Journal	104
Ren et al. [41]	Technological Forecasting and Social Change	77
Naeem et al. [35]	Journal of Cleaner Production	69
Saeed et al. [42]	Energies	57
Dou et al. [15]	Resources Policy	55
Elsayed et al. [16]	Energy Economics	46
Le et al. [27]	Research in International Business and Finance	41

The findings displayed in Table 2 provide further details regarding the scholarly journals that have consistently published key works in the field of global economic policy uncertainty and green bonds. Notably, there is a significant focus on the publications found in “Technological Forecasting and Social Change” and “Energy Economics.” These scholarly publications have evolved as prominent platforms for the dissemination of influential research, as indicated by the substantial number of citations linked with their separate articles. An example of a highly cited publication is the work by Le et al. [28] in the journal “Technological Forecasting and Social Change,” which has received the largest number of citations, totaling 147. Similarly, the scholarly article authored by Yu et al. [57] published in the journal “Energy Economics” has garnered significant attention, as seen by its 140 citations. Hence, scholars in the respective discipline may perceive these academic journals as excellent platforms for publishing their research, given their established reputation for spreading influential findings that contribute to the advancement of knowledge in this intricate and evolving field of inquiry.

#### 3.1.6 Methodologies and approaches in the literature

The methodologies and approaches employed by authors in this systematic literature review indicate a prevalent use of Quantile approaches, with a majority of the papers adopting this method to examine the relationship between global economic policy uncertainty and green bond returns. Quantile approaches offer a robust means to capture and analyze potential variations in the data, providing valuable insights into how economic policy uncertainties affect green bond performance across different quantiles or segments. Furthermore, Table 3 presents a diverse range of additional methodologies and approaches utilized by researchers, suggesting a multifaceted approach to studying this complex intersection.

**Table 3:** List of authors and methods/approaches.

Author	Method/Approach
Sinha et al. [44]	Combination of Quantile-on-Quantile Regression and Wavelet multiscale Decomposition approach
Tiwari et al. [47]	TVP-VAR approach
Adekoya et al. [1]	Feasible Quansi-Generalized least squares and the causality-in-quantile estimators
Haq et al. [20]	DCC-MGARCH model
Peng et al. [37]	Panel fixed effect model
Elsayed et al. [16]	Multivariate wavelet approach
Sohag et al. [45]	Cross-Quantilogram and Quantile and Quantile approaches
Wang et al. [50]	Quantile Autoregressive Distributed Lag model
Chai et al. [10]	TVP-VAR approach
Xu et al. [54]	Comprehensive meta-analysis approach
Haq et al. [21]	Wavelet coherence analysis
Le et al. [27]	ARDL bounds testing approach
Saeed et al. [42]	Corrected dynamic conditional correlation models
Dou et al. [15]	Quantile granger test and Quantile regression methods
Mensi et al. [33]	Copulas, CoVaR, and Quantile regression approaches
Ren et al. [41]	Combination of the maximum overlap discrete wavelet transform and two Quantile methods
Bossman et al. [8]	A set of Parametric and nonparametric Quantile-based techniques
Khalfaoui et al. [25]	Combination of Diebold & Yilmaz's (2012, 2014) time domain spillover approach and Ando et al's (2022) quantile regression framework

## 4 Discussion of results

### 4.1 Research focus on global economic policy uncertainty and green bond

From the results of this systematic literature review, it is evident that researchers have paid increasing attention to the intersection of global economic policy uncertainty and green bond returns. With the substantial growth on the topic, especially from 2021 onward, the surge in research aligns with global events, such as the COVID-19 pandemic in 2020 and the Russia-Ukraine war that began in February 2022, which prompted scholars to explore the relationship between economic policy uncertainty and green bond performance. Additionally, the Keyword Co-occurrence Network Analysis, conducted with the VOSviewer software, also highlights the significant shift in research focus during the study period. With a remarkable increase in publications in 2022 and 2023, it is evident that the research community recognizes the importance of understanding how global economic policy uncertainty influences the dynamics of green bond markets, even in turbulent economic times.

### 4.2 Methodologies

This section examines the most widely used approaches for evaluating the relationship between global economic uncertainty and green bonds, as well as cross-market study. The goal of analyzing research procedures is to show how effective each approach is at measuring the relationship without favoring any of the models employed in the sample. Table 3 is a representative summary of the methodology employed in the papers chosen for review.

#### 4.2.1 Quantile approaches

The prevalent use of Quantile approaches in most of the papers considered in the study demonstrates a strong methodological preference for investigating this relationship. Quantile approaches are well-suited for this topic as they enable researchers to capture and analyze potential variations in the data across different quantiles or segments. Concerning green bond returns, this permits researchers to assess how economic policy uncertainties affect these returns differently under various market conditions. For instance, they can explore whether green bonds perform differently during periods of high policy uncertainty compared to times of relative stability. This approach was utilized by researchers either by modifying the quantile approach or adding other models to deal with some setbacks of the quantile methods. Studies such as [43], [56], [15], [45], [50] all utilized the Quantile approaches to assess these relationships. This method offers a nuanced comprehension of the relationship and aids in pinpointing the specific circumstances in which economic policy uncertainty significantly impacts green bond performance.

Even though the Quantile approaches are very useful in the field, one major setback that should be noted is that, they may not capture the full complexity of the relationship, as they focus on specific quantiles of the distribution, potentially missing important dynamics in the middle of the distribution. Additionally, quantile approaches might not provide a clear understanding of the overall pattern or direction of the relationship, as they only examine conditional quantiles. To address these limitations, researchers can complement quantile analysis with other statistical techniques like regression analysis or time series modeling to obtain a more comprehensive view of the relationship, allowing for a better understanding of the impact of economic uncertainty on green bond returns across the entire distribution. Sinha et al. [44] tackled this limitation by integrating the Quantile-on-Quantile Regression and Wavelet Multiscale Decomposition methods to assess the influence of green bond financing on environmental and social sustainability. Their findings indicated a potential gradual adverse transformation in environmental and social responsibility due to green financing mechanisms. In their empirical study, Hung [23] employed a unique approach that combined quantile on quantile regression and Granger causality in quantiles techniques. The objective was to examine the asymmetric association between green bonds and various conventional assets, such as GSCI Commodity Index, Bitcoin price, Clean Energy Index, S&P 500, and CBOE volatility. The findings of their study indicate that the inclusion of other assets in the analysis enhances the performance of green bonds throughout the period examined. Moreover, this effect is particularly significant in the upper quantiles of the corresponding variables. Yan et al. [56] also used the Quantile Autoregressive Lagged Approach (QARDL) to analyze factors affecting the global green bond markets, such as energy prices, green energy stocks, and gold prices. They also applied the Quantile Granger Causality test to determine the causal connections between these variables. The QARDL analysis confirmed a strong, lasting relationship between these variables and the global green bond market. Moreover, the Granger-causality test revealed a two-way causal link between the global green bond market, energy prices, green energy stocks, and gold prices. Wang et al. [50] conducted a more intriguing study that looked into the effects of the price of crude oil and economic policy uncertainty on China's green bond index using a quantile autoregressive distributed lag model. The empirical results of their research showed that over the long term, crude oil price had a notably positive impact on green bond index across most quantiles, whereas economic policy uncertainty had a largely negative influence. Conversely, in the short term, these effects reversed and were significant only in the higher quantiles. In a recent study by Adekoya et al. [1], the Feasible Quasi-Generalized Least Squares (FQGLS) and causality-in-quantiles methods was utilized to forecast the performance of green bond returns. They discovered that many variables are crucial for predicting green bond returns. Specifically, speculative factors negatively affected predictions, while commodity and financial asset prices had a varied impact on forecasting.

#### 4.2.2 The spillover indexes

The Diebold and Yilmaz [13] index has emerged as a widely utilized tool among the papers considered for this systematic literature review. This index plays a pivotal role in assessing the interconnectedness and dynamic relationships between financial markets. Other notable spillover indexes include [14], [7], [6] indexes, which have also been utilized by literature. Their applications within the reviewed papers signify its value as a comprehensive measure to gauge the spillover effects and transmission of shocks across various markets. These indexes offer a robust quantitative framework for researchers to analyze the extent to which changes in one market can affect others [13], providing critical insights into the interdependencies within the global financial system. Some notable studies that utilized these indexes include [28], [35], [16], [25], [55]

Le et al. [28] conducted a study where they employed both the Diebold and Yilmaz [13] and Baruník et al. [7] methods to investigate how the volatility of returns in Fintech, green bonds, and cryptocurrencies relates to each other during the era of the 4th industrial revolution. The findings from DY indicate two main points. First, there is a significant degree of interconnectedness between 21st-century technology assets and traditional common stocks. This suggests that in times of economic turbulence, the likelihood of simultaneous losses across these assets is quite high. Second, among the assets studied, Bitcoin as well as the MSCI US, MSCIW, and KFTX are sources of volatility shocks, while US dollar, green bonds, green bond select, oil, gold, and VIX tend to absorb these shocks. Another study by Naeem et al. [35] explored how green bonds and commodities are interconnected, both in terms of time and frequency, using the spillover frameworks developed by Diebold and Yılmaz [14], as well as Baruník and Křehlík [6] approach. The study's results indicated that commodities within the same category tended to have stronger spillover effects. Notably, gold and silver consistently displayed the most robust connections with green bonds, regardless of the time periods considered. Interestingly, crude oil demonstrated a pronounced association with green bonds, particularly over longer time horizons. Yadav et al. [55] also employed Diebold and Yilmaz tests, along with Barunik and Krehlic tests, to analyze the interconnections between green bonds and the economies of the top ten European countries. These countries include Austria, Sweden, Germany, Luxembourg, Norway, Denmark, Switzerland, Netherlands, Iceland, and Belgium, all of which are part of the OECD. The research findings revealed varying levels of volatility across different timeframes, encompassing short, medium, and long terms.

#### 4.2.3 VAR models

Other methods employed by studies in the review include VAR models. These approaches were utilized by studies like [47], [10], [2], [31]. VAR models, especially Time-Varying Parameter Vector Autoregression Model (TVP-VAR model) help establish causal links, forecast future values, and conduct impulse response analysis, shedding light on how policy uncertainty affects green bond returns and vice versa. Their insights are valuable for investors and policymakers, aiding in informed decision-making and policy design to promote sustainability and economic stability. For example, Tiwari et al. [47] utilized the TVP-VAR methodology to explore the interplay of return influences and associations within the price indices of S&P Green Bond, Solactive Global Wind, S&P Global Clean Energy, Solactive Global Solar, and Carbon. The findings from the TVP-VAR analysis revealed that the overall interconnection dynamics among these assets exhibited variations over time and were influenced by economic events. Chai et al. [10] also employed the (TVP-VAR) to investigate the ever-changing connections between green bonds, clean energy, and stock prices during the global market's response to the COVID-19 pandemic. Their findings revealed that, leading up to the pandemic, the influence of clean energy on stock prices grew steadily. Furthermore, their analysis of impulse responses at various time horizons suggested that green bonds initially caused a short-term boost in clean energy, with this positive effect intensifying post-COVID-19 outbreak. Interestingly, the pandemic also lessened the adverse impact of green bonds on stock prices in the medium term. Lastly, by examining impulse responses at different points in time, the study revealed that stock prices tended to rise following a positive shock to clean energy, with this effect being particularly pronounced during economic recovery periods compared to other periods. In their study, Tsagkanos et al. [49] delved into the correlation between corporate green bonds and a diverse array of commodities. Their work contributes to our comprehension of the enduring dynamics between green bonds and commodities. The study employed an innovative methodology, utilizing VaR-based copulas, to enhance the analysis by capturing asymmetric risk spillover and accounting for the asymmetric tail distribution. One notable outcome of the study is the identification of an insignificant risk spillover effect from commodity market uncertainty. This implies that, at least within the examined context, fluctuations in commodity markets do not significantly affect the risk associated with corporate green bonds. However, the study uncovered an intriguing relationship between non-perishable and perishable commodities, with the former transmitting risk to the latter, excluding lead. Additionally, the research emphasized the relatively higher risk spillover in specific commodities like lead, gold, and agriculture, in contrast to copper and silver, which exhibit lower risk spillover. Energy commodities, on the other hand, displayed the least spillover effect.

#### 4.2.4 GARCH family models and other approaches

Other studies included in the review have also made use of GARCH models and various alternative methodologies. Haq et al. [20] utilized the DCC-MGARCH model to examine the dynamic interrelationships between global rare earth elements, green bonds, clean energy stocks, and economic policy uncertainty. They reported that, when economic policy is uncertain, green bonds typically act more as a hedge than as a haven. Marín-Rodríguez et al. [32] also employed the same methodology (DCC-Garch) Model and also included the Granger Causality Test to examine the relationship between green bonds, CO2 futures' prices, and oil prices. Their analysis focused on extreme market conditions such as the COVID-19 pandemic and the Russian invasion of Ukraine. The results revealed that the Green Bond Index had a negative dynamic correlation with oil returns and CO2 futures' returns, particularly during uncertain periods. To investigate how Economic Policy Uncertainty (EPU) affects the carbon futures market under different market conditions, Dou et al. [15] used both the quantile Granger test method and the quantile regression method in their study. They also employed the wavelet decomposition approach to examine how the spillover from EPU to carbon futures price returns evolves over time and across different frequency domains. The findings of their study revealed that EPU shocks do not have the ability to predict the daily volatility of carbon futures returns. However, in the long run, EPU has a notable negative impact on carbon futures price returns. Another study by Mensi et al. [33] employed various models, such as Diebold and Yılmaz [14], the TVP-VAR model, and Baruník and Křehlík [6] frequency spillover index to analyze how the dynamics of spillovers in returns and volatility played out, along with assessing the hedging effectiveness of assets like Green Bonds, oil, gold, the US dollar index, silver, and volatility index against declining US stock prices. They examined both the period before and during the COVID-19 pandemic, covering both short-term and long-term scenarios. The results they obtained indicated that short-term volatility spillovers were more pronounced than their long-term counterparts. Furthermore, Green Bonds emerged as net transmitters of spillovers in the short term but shifted to net receivers in the long term.

#### 4.2.5 Conclusions on the various methodologies employed

The economic significance of this research centers on comprehending the impact of variations and uncertainties in global economic policies on green bond returns. Green bonds play a crucial role in promoting environmentally sustainable projects. Hence, this study provides insights into how uncertainties in economic policies, whether arising from geopolitical events, regulatory changes, or economic crises, influence the financial performance of green bonds. From our review, one observation is shared by all of the approaches employed in the literature. In particular, when there are major global economic events, the relationship between the uncertainty of the global economic policy and the returns on green bonds is more obvious. The effects of global economic policy uncertainty on green bonds, however, are temporary. The studies together highlight the complex nature of the link between uncertainty in global economic policy and returns on green bonds, showing that the relationship is influenced by a variety of dynamic elements such as financing structures, market dynamics, and outside economic effects. These results highlight the need for a detailed and thorough study of the market dynamics for green bonds. In addition, they emphasize the wider connections that exist between green bonds and other financial assets, like commodities and assets related to 21st-century technology. This emphasizes the necessity for investors to understand the complex relationship between economic policy uncertainty and the performance of green bonds across a range of scenarios and time periods in order to make well-informed investment decisions.

## 5 Conclusion and recommendations

This study involved a systematic review of the connection between recently published green bond and global economic policy uncertainty research spanning from January 2016 to September 2023. The review shows that researchers have increasingly examined the connection between global economic policy uncertainty and green bond returns. This rise in research, particularly from 2021 onward, corresponds with major global events like the COVID-19 pandemic in 2020 and the Russia-Ukraine conflict that started in February 2022. These events prompted scholars to investigate how economic policy uncertainty affects green bond performance.

Regarding the contributions on this topic by different countries, China has the highest contribution, followed by the UK, Vietnam, and Pakistan. Additionally, the review highlights common methodologies used to assess the relationship between global economic policy uncertainty and green bonds. These methodologies encompass Quantile approaches, the Diebold and Yılmaz [14], TVP-VAR models, GARCH models, ARDL models, as well as others such as the Wavelet approaches and the Baruník and Křehlík [6] spillover index.

Numerous authors have shown that green bonds can serve as a hedge against economic policy uncertainty. In the current body of research, multiple studies have concluded that the connection between global economic policy uncertainty and green bonds becomes more pronounced during periods of global uncertainty. Nevertheless, some studies argue that the impact of global economic policy uncertainty on green bonds is short-term and is mainly a result of spillover effects. This means that the influence on certain financial assets spills over to affect green bonds, which tend to function more as a hedge than a safe haven. These collective studies underscore the intricate nature of the relationship between uncertainty in global economic policy and the returns on green bonds, revealing that this relationship is influenced by various dynamic factors such as financing structures, market dynamics, and external economic forces.

There are certain limitations to this study that may be overcome in future research endeavors. To begin with, the study's sample was drawn exclusively from a single database, Scopus, and was restricted to research articles published in English language. This means there is a likelihood that publications from other databases and those in languages other than English were inadvertently omitted from consideration. It is also imperative to acknowledge the temporal constraints imposed on this study, limiting the scope to research publications released between January 2016 and September 2023. While this timeframe allows for a comprehensive examination of the existing literature, it is crucial to recognize the possibility of excluding pertinent studies published both before and after this defined period. Additionally, the primary focus of the study is to investigate the impacts of economic policy uncertainty on green bond returns. While this focus is to provide a targeted analysis, we acknowledge the influence of other potential factors. Recognizing this, we understand the importance of examining a wider range of factors in future research to achieve a more thorough understanding of the dynamics surrounding green bonds. The emphasis on economic policy uncertainty in the current investigation is a foundational step, and our commitment extends to exploring additional factors that may affect the relationship between green bonds and various economic elements in subsequent studies.

### 5.1 Recommendations for future research

Table 4 outlines a few potential avenues for research that may be investigated subsequent to this review.

**Table 4:** Prospective research avenues and some useful papers.

	Research Direction	Useful papers
1.	Compare the performance of green bonds with other financial assets, including traditional bonds, commodities, and assets related to 21st-century technology. This analysis can shed light on the distinctive features and vulnerabilities of green bonds in the context of economic policy uncertainty.	(Le et al. [28], Wang et al. [50], Tiwari et al. [48])
2.	Examine how factors such as investor sentiment, regulatory changes, and market liquidity influence the performance of green bonds during times of economic uncertainty.	(Piñeiro-Chousa et al. [39], Wei et al. [52])
3.	Explore the enduring effects of global economic policy uncertainty on green bonds. Assess whether specific events or prolonged periods of uncertainty have a lasting impact on green bond performance and evaluate the sustainability of these effects.	(Wang et al. [50], Zhong et al. [60])
4.	Explore the potential of machine learning algorithms in assessing the impacts of various variables on green bond returns.	(Chen et al. [11], Mohsin and Jamaani [34], Gyamerah et al. [19])

## Funding

The authors did not receive funding for the study.

## CRediT authorship contribution statement

**Samuel Asante Gyamerah:** Writing – review & editing, Validation, Supervision, Conceptualization. **Clement Asare:** Writing – original draft, Visualization, Software, Methodology, Formal analysis.

## Declaration of Competing Interest

The authors declare that they have no known competing financial interests or personal relationships that could have appeared to influence the work reported in this paper.

## Data Availability

No data was used for the research described in the article.
